# Comparison of efficacy between internal fixation of minimally invasive elastic stable intramedullary nail and plate in the treatment of pediatric femoral shaft fracture

**DOI:** 10.12669/pjms.35.5.513

**Published:** 2019

**Authors:** Wenxia Wang, Xiaoyong Zheng, Zuoyong Sun

**Affiliations:** 1 Wenxia Wang Department of Pediatric Surgery, Binzhou People’s Hospital, Shandong, 256600, China; 2 Xiaoyong Zheng Department of Cardiothoracic Surgery, Binzhou People’s Hospital, Shandong, 256600, China; 3 Zuoyong Sun Department of Cardiothoracic Surgery, Binzhou People’s Hospital, Shandong, 256600, China

**Keywords:** Femoral shaft fracture, Internal fixation of minimally invasive elastic stable intramedullary nail, Plate internal fixation

## Abstract

**Objective:**

To compare and analyze the clinical effects of internal fixation of minimally invasive elastic stable intramedullary nail and plate in the treatment of pediatric femoral shaft fracture.

**Methods:**

A total of 120 children with femoral shaft fractures who were admitted to our hospital from December 2016 to April 2018 were enrolled. The children were divided into an observation group and a control group by random number table, with 60 children in each group. The children in the observation group underwent internal fixation of minimally invasive elastic stable intramedullary nail, while those in the control group underwent open reduction based on internal fixation of plate. The surgical status and postoperative complications of the two groups were observed and compared, and Kolmert knee function scoring criteria were used for assessing the surgical effects of children.

**Results:**

The operation duration, intraoperative blood loss, hospitalization duration, fracture healing time and time of off-bed loaded activity of the observation group were significantly shorter than those of the control group, and the differences were statistically significant (P<0.05). The excellent and good rate of fracture healing in the observation group was 100%, which was higher than that of the control group, 83.33%, and the difference was statistically significant (P<0.05). The total incidence rate of complications in the observation group was 8.33%, which was lower than that of the control group, 10.00%, but the difference was not statistically significant (P>0.05).

**Conclusion:**

Pediatric femoral shaft fractures can be treated with internal fixation of minimally invasive elastic intramedullary nail, and it has advantages of significant curative effect, small trauma and fast postoperative recovery, which is conducive to fracture healing and worth promoting.

## INTRODUCTION

Femoral shaft is the longest tubular bone in the human body, and strong direct violence often causes femoral shaft fracture.^1^,^2^ The incidence rate of femoral shaft fracture among males is higher than that of females. It often occurs to young and middle-aged groups and sometimes happens in the pediatric group due to car accidents, accidental injuries, etc. Pediatric femoral fracture is a common type of pediatric fracture, and statistics showed that the prevalence of pediatric femoral fracture was about 10% to 15%,^3^ and they could be divided into subtrochanteric fracture, femur proximal 1/3 fracture, femur 1/3 fracture, femur 1/3 distal fracture and supracondylar fracture according to the fracture locations, among which femur 1/3 fracture is the most common one, with an incidence of 70%. If child with femoral shaft fracture fails to receive effective treatment in time, complications like lower limb deformity may occur.^4^ Pediatric femoral shaft fracture is usually closed injuries; previously they were often treated with traction and reduction, plaster immobilization and so on, but the course of the treatment was long, which might have a more serious negative impact on quality of life and growth of children.^5^,^6^ In recent years, with the continuous changes in the social traffic environment and living environment, the incidence of pediatric femoral shaft fracture continues to increase, which requires higher on therapies and clinical efficacy. Internal fixation of minimally invasive elastic stable intramedullary nail has been widely used in the treatment of pediatric femoral shaft fracture over these years, and compared with the traditional internal plate fixation method, the advantage of elastic stable intramedullary nailing is minimally invasive.^7^,^8^

In this study, 120 children with femoral shaft fractures who were admitted to our hospital were selected as subjects, The therapeutic effects of closed reduction based on elastic intramedullary nailing internal fixation and open reduction based on internal fixation of plate in the treatment of femoral shaft fracture were compared, and the indications, advantages and disadvantages of the two treatment methods were analyzed, aiming to provide a reference for the clinical treatment schedules.

## METHODS

A total of 120 children with femoral shaft fractures who were admitted to the hospital from December 2016 to April 2018 were selected. The inclusive criteria included being diagnosed with closed fractures by X-ray examination, without nerve and vascular injuries, and fracture time less than seven days. The exclusive criteria included comminuted fractures, open fractures, endocrine diseases, mental disorders, severe organ diseases and surgical contraindications. All the children were divided into an observation group and a control group according to the random number table method, 60 each group. The study was approved by the ethics committee of our hospital, and all the guardians of children signed informed consent.

In the control group, children were treated with internal fixation of plate. Firstly, the patients received general anesthesia. They were disinfected, and then a surgical incision was made around the fracture end to expose the fracture end. Then subperiosteal stripping was performed for fracture reduction. A locking compression plate with a proper length was placed at the external side of the shaft for fixation. The incision was closed. Negative pressure drainage was inserted, and finally the wound was bound.

In the observation group, patients received internal fixation of minimally invasive elastic stable intramedullary nail. General anesthesia was performed. Under the guidance of C-arm X-ray machine, the anteroposterior and lateral fracture end was observed. Reduction was given to the fracture site using closed reduction method. Intramedullary needle of the proper type was selected according to the actual situations. The needle was punctured at the site where was 1 ~ 2 cm away from the distal epiphysis of the femur and then inserted into the medullary space. A bone awl was vertically penetrated to the medullary space, slowly rotated, and pushed to make the intramedullary needle bend. The other intramedullary needle was inserted into the medullary space in the same way at the opposite site, and a bone awl was also inserted to make the needle bend. The two needles were fixed well, and their tails were cut off. The segment of the needles outside the body was bent and embedded under the skin.

Children in the two groups were given antibiotics for two days and nursing intervention. The first intervention was mental nursing. The children were visited the day before surgery. The medical record was carefully read, and the examination results were checked to evaluate the conditions of blood vessels, skin and nutrition. Moreover initial emotion exchange was performed, and children in different age grades were given different forms of praise and encouragement to relieve the children’s fear of surgery. The second intervention was position nursing. The children took a supine position on the traction table. The nurses assisted doctors to adjust the position of children; they held the affected limb gently with both hands. The nurses should ensure that the position made the children feel comfortable, had no severe influence on respiration and circulation, and fully exposed the operating field.

### Observation indicators

The relevant surgical indicators of the patients in the two groups, including the operation duration, intraoperative blood loss, hospitalization duration, fracture healing time, and time of off-bed loaded activity, were compared.

The surgical efficacy of patients was compared between the two groups. The Kolmert knee function score criteria were used for evaluating the fracture healing of children.^9^ The excellent and good rate could be calculated using the formula: excellent and good rate = (number of excellent cases + number of good cases) / total number of cases × 100%. The postoperative complications of patients were compared between the two groups.

### Statistical Analysis

Data were analyzed by SPSS ver. 21.0. Measurement data were expressed as mean±SD and processed by t test; enumeration data were expressed by percentage (%) and processed by Chi-square test. P<0.05 meant that difference was statistically significant.

## RESULTS

There were 38 males and 22 females in the observation group; they aged 4 to 15 years old (average 10.36±2.19 years old); 35 cases were caused by traffic accidents, 15 by high falls, and 10 by other injuries. As to Sein-sheimer classification of fractures, there were 40 cases of Tyupe-II, 10 cases of Type-III and 10 cases of Type-IV. There were 36 males and 24 females in the control group; they aged 4 to 14 years old (average 6.55±2.06 years old); 39 cases were caused by traffic accidents, 16 by high falls, and 5 by other injuries. As to Sein-sheimer classification of fractures, there were 27 cases of Type-II, 14 cases of Type-III and 19 cases of Type-IV. There was no significant difference in the clinical data between the two groups (P>0.05); therefore the results were comparable.

The operation duration, intraoperative blood loss, hospitalization duration, fracture healing time, and time of loaded off-bed activity of the observation group were significantly shorter than those of the control group, and the differences were statistically significant (P<0.05, Table-I).

**Table I:** Comparison of surgical indicators between the two groups (Mean±SD).

Group	Observation group (n=60)	Control group (n=60)	t	P
Operation duration (min)	68.93±7.18	142.35±14.28	37.61	<0.05
Intraoperative blood loss (mL)	61.29±6.26	142.16±14.92	40.93	<0.05
Hospitalization duration (d)	6.78±0.43	15.27±1.32	39.62	<0.05
Fracture healing time (d)	62.72±6.36	80.55±7.28	15.12	<0.05
Time of loaded off-bed activity (d)	82.18±8.25	125.72±12.37	23.97	<0.05

**Fig.1:**
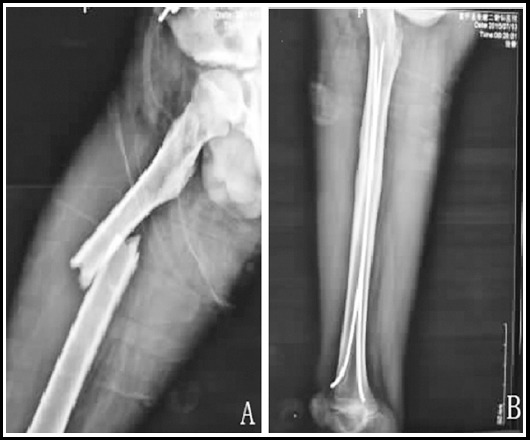
A 9-year-old child with the right upper femoral transverse fracture ***Note:*** A is the preoperative X-ray film; B is the X-ray film 5 months after surgery.

After treatment, the excellent and good rate of fracture healing in the observation group was higher than that in the control group (P<0.05, Table-II). Fig.1 shows X-ray films before surgery and five months after surgery of minimally invasive intramedullary nailing in the right upper femoral transverse fracture of a child, which indicated good healing.

**Table II:** Comparison of fracture healing between the two groups [n(%)].

Group	Observation group (n=60)	Control group (n=60)	X^2^	P
Excellent	37(61.67)	12(20.00)	6.469	<0.05
Good	23(38.33)	38(63.33)		
Moderate	0(0.00)	10(16.67)		
Poor	0(0.00)	0(0.00)		
Excellent and good rate	60(100.00)	49(83.33)		

The total incidence of complications in the observation group was 8.82%, which was lower than that in the control group, 9.09%, but the difference was not significant (P>0.05, Table-III).

**Table III:** Occurrence of complications of the two groups [n(%)].

Group	Observation group (n=60)	Control group (n=60)	X^2^	P
Skin infection	2(3.33)	0(0.00)	0.0007	>0.05
Ankylosis	1(1.67)	2(3.23)		
Angulation deformity	2(3.33)	4(6.67)		
Overall incidence	5(8.33)	6(10.00)		

## DISCUSSION

With the continuous improvement of medical technology, there are more and more clinical methods for treating femoral shaft fractures, but at present, there is no unified conclusion on how to treat pediatric femoral shaft fracture.^10^,^11^ Previously, it was often recommended to implement conservative treatment for children. However, clinical investigations have found that conservative treatment took longer time and longer hospitalization time. Most of the children were immature and prone to poor treatment compliance. Therefore, some scholars believed that surgery could be performed on children.^12^,^13^ In the early stage, external fixation with plaster was used, but the fixation strength was poor. Children were often unwilling to cooperate, which might result in different degrees of displacement of fracture ends. In a long term, complications such as malunion or joint stiffness and skin pressure sore might occur, leading to a poor curative efficacy and poor quality of living.^14^ In recent years, internal fixation has become the mainstream treatment method for clinical fracture, among which elastic stable intramedullary nailing and plate internal fixation are the most widely used.^15^,^16^

In this study, children with femoral shaft fracture were treated with elastic stable intramedullary nailing and conventional plate fixation. The study found that the conventional plate fixation treatment had characteristics of longer length of surgical incision and more intraoperative bleeding although it was effective, and it had a negative impact on the prognosis of children due to the poor compliance of children. Compared with the conventional plate fixation, internal fixation of elastic stable intramedullary nail has favourable elastic restoring force as its main material is titanium alloy, which is more helpful to the stability after fracture reduction and has a positive effect for the recovery of physiological curvature of shaft of femur.^17^,^18^ Moreover it can resist the tension, pressure and rotating force at the fracture site, which is more conductive to generation of callus and can promote the early healing of fracture.^19^

The results of this study showed that the excellent and good rate of children in the observation group was 100.00%, which was significantly higher than that of the control group, 83.33%, and the operation duration and intraoperative blood loss in the observation group were significantly less than those in the control group, which was consistent with the research results of Govindasamy et al.^20^ It suggested that elastic stable intramedullary nailing in the treatment of femoral shaft fractures had better therapeutic efficacy and smaller surgical trauma compared with the conventional plate fixation. Moreover the results also showed that the hospitalization duration, fracture healing time and time of off-bed loaded activity of the observation group were significantly shorter than those of the control group, which was consistent with the previous research results.^21^,^22^ It indicated that internal fixation of elastic stable intramedullary nail accelerated the postoperative recovery of children and was more effective in improving quality of life of children.

## CONCLUSION

The application of elastic stable intramedullary nailing has good therapeutic effect and fewer complications in the clinical treatment of pediatric femoral shaft fracture, which can effectively promote the healing of fracture sites and help children to recover as soon as possible. It is worth clinical application.

### Authors’ Contribution

**WXW:** Study design, data collection and analysis.

**WXW & XYZ:** Manuscript preparation, drafting and revising.

**WXW & ZYS:** Review and final approval of manuscript.
